# The complete mitochondrial genome of *Arius arius* (Siluriformes: Ariidae)

**DOI:** 10.1080/23802359.2016.1198999

**Published:** 2016-12-23

**Authors:** Pengfei Wang, Youjun Ou, Jiufu Wen, Jia’er Li

**Affiliations:** Key Laboratory of South China Sea Fishery Resources Exploitation & Utilization, Ministry of Agriculture, South China Sea Fisheries Research Institute, Chinese Academy of Fishery Sciences, Guangzhou, China

**Keywords:** Ariidae, *Arius arius*, mitochondrial genome, Siluriformes

## Abstract

The threadfin sea catfish (*Arius arius*) belongs to the genus *Arius* in Ariidae. In this paper, we initially determined the complete mitochondrial genome of *Arius arius*. The mitochondrial genome is 16, 711 bp in length, with the base composition on the heavy strand: A – 29.67%, T – 25.38%, C – 29.70% and G – 15.25%. It has the typical vertebrate mitochondrial gene arrangement, including 13 protein-coding genes, 22 *tRNA* genes, 2 *rRNA* genes and a control region. Phylogenetic analysis showed that *A. arius* was clustered into the order of Siluriformes, and closely related to species in the family of Siluridae. The present study would contribute to genetic resources conservation and systematics study of *A. arius*.

The threadfin sea catfish (*Arius arius*) is found in marine and brackish water with distribution in the South China Sea and Indo-west Pacific (Wang et al. 2005; Marceniuk & Menezes 2007). *Arius arius* distribution has been considered to be declining across the Indo–Malaysian archipelago sea waters (Ng 2012), and it has been scheduled on the IUCN Red List of threatened species (Pal 2012). Mitochondrial genome has been used as a helpful tool for molecular taxonomy, identification and study of the population structure and dynamics due to its specialities of mitochondrial inheritance, neutral evolution and clock-like evolutionary rate (Galtier et al. 2009; Hu et al. 2015; Li et al. 2015). However, so far, no complete mitogenome was available in the family of Ariidae that includes around 150 species, which restrict related researches. Therefore, in this study, the complete mitogenome of *A. arius* will be characterized and phylogenetically analyzed. We expect that the genomic data will provide essential information to genetic resources conservation and systematics study for *A. arius*.

*Arius arius* was collected from the South China Sea (21°88′N, 113°18′E). Muscle was sampled and frozen in liquid nitrogen and then stored at −80 °C in South China Sea Fisheries Research Institute, China. The mtDNA was extracted using the Mitochondrial DNA Isolation Kit (Haling Biotech Shanghai, Co., Ltd, China). Illumina sequencing was performed on Illumina HiSeq Sequencing System (Illumina Inc.) using 2 × 125 bp chemistry kit. A total of 2.02 Gbp clean data were assembled by the SOAPdenovo Assembler program (v. 2.04) and GapCloser (v.1.12) (Beijing Genomics Institute, Shenzhen, China), and the assembled mtDNA was checked by PCR.

The complete mitogenome of *A. arius* (GenBank accession: KX211965) is a closed-circular molecule of 16,711 bp with the base composition on the heavy strand: A – 29.67%, T – 25.38%, C – 29.70% and G – 15.25%. It presents the typical set of 37 genes observed in metazoan mitogenomes, including 13 protein-coding genes (*ND1-6*, *ND4L*, *COI-III*, *Cytb*, *ATP6* and *ATP8*), 22 *tRNA* genes (one for each amino acid, two each for Leucine and Serine), and 2 *rRNA* genes (*12S rRNA* and *16S rRNA*). The organization of *A. arius* mitogenome conforms to the consensus of gene order of that in other fish species. Twelve of the 13 protein-coding genes use ATG as start codon, with only *COI* using the GTG codon. Six open reading frames end with TAA (*ND1*, *ND4L*, *ND5*, *COI*, *ATPase 6* and *ATPase8*), five genes use incomplete stop codon as T– (*ND3*, *ND4*, *COII*, *COIII* and *Cytb*), and two genes use TAG as stop codon (*ND2* and *ND6*). Twenty-two *tRNA* genes are scattered throughout the mitogenome, and vary from 66 to 75 bp and display a typical clover-leaf secondary structure, except for *tRNA^Ser(AGN)^*. The control region of 1077 bp, with high A + T content (62.67%), is located at the conserved position between *tRNA^Pro^* and *tRNA^Phe^*. According to the phylogenetic tree (Figure 1), as the first mitogenome acquired in Ariidae, *A. arius* is clustered into the group of Siluriformes, and closely related to species in Siluridae, and divided from other families.

**Figure 1.**
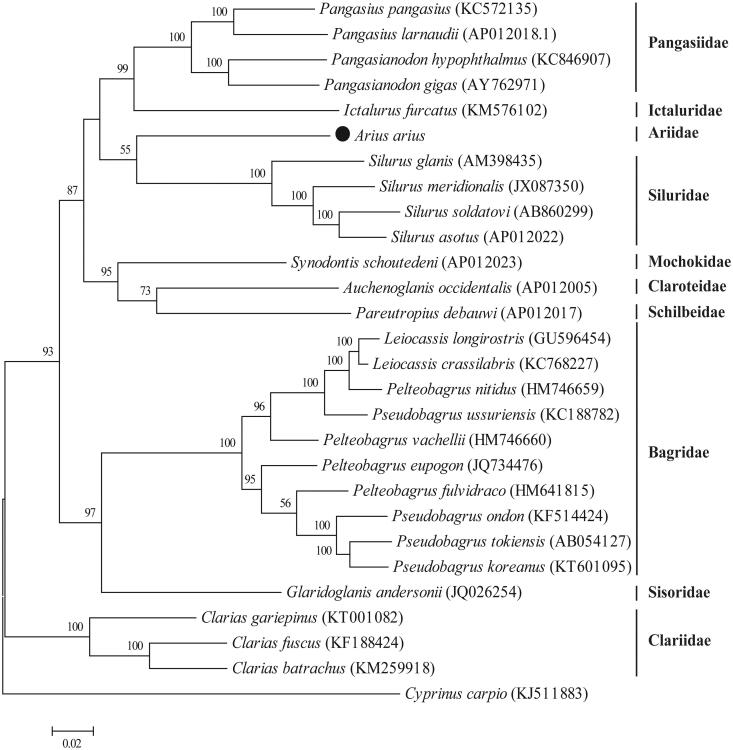
Phylogenetic analysis of *Arius arius* and other fishes in the order of Siluriformes. The complete mitochondrial genomes were compared using the maximum-likelihood method with MEGA 6.0 software (Tamura et al., 2013). The tree with the highest log likelihood (155,680.29) is shown. Bootstrap support values (1000 replicates) are indicated at the nodes.
